# Genetic Diversity, Morphological Uniformity and Polyketide Production in Dinoflagellates (*Amphidinium*, Dinoflagellata)

**DOI:** 10.1371/journal.pone.0038253

**Published:** 2012-06-04

**Authors:** Shauna A. Murray, Tamsyn Garby, Mona Hoppenrath, Brett A. Neilan

**Affiliations:** 1 School of Biotechnology and Biomolecular Sciences and Evolution and Ecology Research Centre, University of New South Wales, New South Wales, Sydney, Australia; 2 Sydney Institute of Marine Sciences, Mosman, New South Wales, Australia; 3 Forschungsinstitut Senckenberg, Deutsches Zentrum für Marine Biodiversitätsforschung (DZMB), Wilhelmshaven, Germany; University of Lausanne, Switzerland

## Abstract

Dinoflagellates are an intriguing group of eukaryotes, showing many unusual morphological and genetic features. Some groups of dinoflagellates are morphologically highly uniform, despite indications of genetic diversity. The species *Amphidinium carterae* is abundant and cosmopolitan in marine environments, grows easily in culture, and has therefore been used as a ‘model’ dinoflagellate in research into dinoflagellate genetics, polyketide production and photosynthesis. We have investigated the diversity of ‘cryptic’ species of *Amphidinium* that are morphologically similar to *A. carterae*, including the very similar species *Amphidinium massartii*, based on light and electron microscopy, two nuclear gene regions (LSU rDNA and ITS rDNA) and one mitochondrial gene region (cytochrome *b*). We found that six genetically distinct cryptic species (clades) exist within the species *A. massartii* and four within *A. carterae*, and that these clades differ from one another in molecular sequences at levels comparable to other dinoflagellate species, genera or even families. Using primers based on an alignment of alveolate ketosynthase sequences, we isolated partial ketosynthase genes from several *Amphidinium* species. We compared these genes to known dinoflagellate ketosynthase genes and investigated the evolution and diversity of the strains of *Amphidinium* that produce them.

## Introduction

Dinoflagellates are a unique group of microbial eukaryotes that play a variety of important ecological roles, notably as the core of aquatic food webs, in symbioses with invertebrates such as corals, and as the agents responsible for producing harmful algal bloom toxins (HABs). While eukaryotic, they possess many characteristics not seen in typical eukaryotes, such as a fifth base replacing uracil in their DNA [1], [2], unusually large genomes, greatly reduced chloroplast genomes [3], permanently condensed chromosomes lacking in histones [2], and complex organelle structures such as eyespots [4]. The use of molecular genetic sequencing to study biodiversity, based primarily on regions of the ribosomal RNA (rRNA) operon, has shown that high levels of genetic diversity exist within morphologically indistinguishable species of dinoflagellates [5], [6]. Moreover, this may be an underestimation of the true diversity present in the group, as 18 s rRNA genes have been found to be more conserved, compared to entire genomes, in unicellular organisms than they are in multicellular organisms [7].

*Amphidinium* Claparède et Lachmann is a widespread genus of dinoflagellate, found in temperate and tropical marine waters, in both free-living benthic and endosymbiotic states. *Amphidinium* species are often amongst the most abundant dinoflagellates in benthic ecosystems [8], and species such as *Amphidinium carterae* Hulburt grow well in culture and are often present in culture collections. For this reason, *A. carterae* has been used as a ‘model dinoflagellate’ in breakthrough studies of the dinoflagellate plastid including the peridinin-chloroplast A-protein light-harvesting antenna complex [9]–[12], the unique dinoflagellate genome [13], the first successful genetic transformation of a dinoflagellate [14] and the first polyketide synthase gene cluster from a dinoflagellate [15].

*Amphidinium* is considered to be a member of the family Gymnodiniaceae, as species lack cellulosic material in their amphiesmal vesicles. However, several molecular phylogenetic studies do not support either the monophyly of the Gymnodiniaceae [16] or a close relationship between *Amphidinium* and other genera of Gymnodiniaceae, such as *Gymnodinium* [17]. *Amphidinium* may be a relatively early evolving lineage of dinoflagellates based on phylogenetic studies of rRNA [17]–[20]. This genus was redefined based on more stringent morphological criteria [17], [18], and now includes approximately 20 known species [21]. Despite the apparent morphological uniformity and simplicity of species of this genus as redefined [18], there may be a very high level of genetic diversity within taxa of this genus, with an intrageneric variation of up to 37% in the unambiguously aligned sequences of the D1–D6 regions of the LSU rDNA [17]. This is a level greater than that of most dinoflagellate orders, and indicates that members of the genus either have comparatively faster evolutionary rates in their rRNA genes than other dinoflagellates, or that they are a very diverse, ancient group. Some species of *Amphidinium* have been reported to possess scales on their cell surface [22], a rare feature amongst dinoflagellates, otherwise only seen in *Oxyrrhis marina* [23], a close relative of dinoflagellates, species of *Heterocapsa* [24], and the prasinophyte-possessing species, *Lepidodinium viride* [25]. Given the high level of genetic diversity found in studies of species of *Amphidinium*, the aim of this study was to examine novel strains of the ‘lab rat’ dinoflagellate *Amphidinium carteae* and the closely related species *Amphidinium massartii* using nuclear (ITS, LSU rRNA) and mitochondrial (cytochrome *b*) gene markers, and light and scanning electron microscopy, in order to determine whether cryptic species may be present.

A second aim of this study was to examine the potential for polyketide production in the examined strains. A large number of toxic polyketide compounds have been characterised from dinoflagellates, including those responsible for harmful algal blooms [21], [22]. As *Amphidinium* species are morphologically relatively uniform, the vast majority of studies of polyketide production in species of this genus have been conducted with unidentified strains (e.g. [26]), hampering efforts to understand the distribution, evolution and diversity of polyketide synthesis in *Amphidinium*. Given their cosmopolitan distribution and the potential for exploitation of the polyketide production of *Amphidinium* species, in this study we assessed the potential for polyketide production, as evidenced by the presence of protistan polyketide synthase genes, in strains that we have characterised based on morphological and molecular markers.

## Methods

### Culture Growth and Maintenance

*Amphidinium* species and strains were obtained from the Australian National Algae Culture Collection (Table 1). Cultures were grown in 20 ml of F/2 [27] or GSe [28] media in 25 cm^2^ tissue culture flasks. Cultures were kept in a light cabinet at 19°C, with a 12/12 light/dark cycle. Cells from dense cultures were collected by centrifugation, the media was removed, and pellets stored at −20°C until use.

**Table 1:** Strains of *Amphidinium* species used in this study.

*Amphidinium* species	Strain number	Place of culture isolation
*Amphidinium carterae*	CS-21	Halifax, Canada
*Amphidinium carterae*	CS-383	Bicheno, TAS, Australia
*Amphidinium carterae*	CS-212	Bay of Naples, Italy
*Amphidinium thermaeum*	CS-109	Coral Sea, Australia
*Amphidinium massartii*	CS-259	Kurrimine Beach, QLD, Australia
*Amphidinium carterae*	CS-740	Port Botany, NSW, Australia

### DNA Isolation

DNA was extracted from approximately 20 µg of frozen cell pellets. To lyse cells, 500 µl CTAB buffer, containing 1% β-mercaptoethanol, was added to the pellet, which was then heated for 1 hour at 65°C, with vortexing approximately every 15 min. Following cell lysis, 500 µl of 24∶1 chloroform:isopropanol was added, and tubes centrifuged at 15 000 rcf for 10 min at 4°C. The water phase was removed, another 500 µl of 24∶1 chloroform:isopropanol added, then centrifuged again at 15 000 rcf for 10 min at 4°C. The water phase was removed, and 1.5 volumes of 96% ethanol and 0.1 volumes of 3 M sodium acetate were added. DNA was left to precipitate overnight at −20°C. DNA was recovered by centrifugation at 15 000 rcf for 10 min at 4°C, and the supernatant removed. The pellet was washed with 200 µl of 70% ethanol, centrifuged again at 15000 rcf for 10 min at 4°C, the supernatant removed, and pellet left to air dry. DNA was then re-dissolved in 30 µl of distilled water. DNA concentrations were checked by Nanodrop (Thermoscientific, USA), and were generally between 500–1000 ng/µl.

### Primer Design

Ketosynthase (KS) domain primers targeted to dinoflagellates were designed based on published *Karenia brevis* KS sequences [29], and other dinoflagellate ESTs found through tBLASTx searches of *Alexandrium catenella* and *Karlodinium micrum* EST libraries [30], recognised as they contained the KS domain conserved amino acid regions and active site residues. A nucleotide alignment of this limited number of available sequences was used to design degenerate primers that amplified a KS domain fragment (Table 2). Additionally, novel primers were designed based on published primers to amplify the complete ITS1-5.8s-ITS2 region (Table 2).

**Table 2:** Primers used in this study.

Primer name	Sequence 5′-3′	Amplifies	Reference
DKSF1	GCATGACGATSGAYACHGCWTGCTC	KS region	This study
DKSF2	AATCARGAYGGVCGMWSYGC	KS region	This study
DKSR1	CTTCTCCTGCGAAGGDCCRTTBGGYGC	KS region	This study
DKSR2	GTCTCCAAGCGADGTKCCMGTKCCRTG	KS region	This study
DKSR3	GCATTCGTBCCRSMRAAKCCRAA	KS region	This study
D1R	ACCCGCTGAATTTAAGCATA	LSU rRNA	[66], [67]:
D3B	TCGGAGGGAACCAGCTACTA	LSU rRNA	[66], [67]:
ITSfor	TTTCCGTAGGTGAACCTGCGG	ITS rRNA	This study, modified from [68] and [69],
ITSrev	ATATGCTTAAATTCAGCGGGT	ITS rRNA	
Dinocob4F	AGCATTTATGGGTTATGTNTTACCTTT	Cytochrome *b*	[29]
Dinocob3R	AGCTTCTANDGMATTATCTGGATG	Cytochrome *b*	[29]

### PCR and Sequencing

Typical cycling conditions for amplification reactions consisted of an initial denaturing step of 94°C for 2 min, followed by 35 cycles of 94°C for 20 s, 56°C for 30 s, and 72°C for 1 min, followed by a final extension step of 7 min. PCR products were separated by agarose gel electrophoresis, then stained with ethidium bromide and visualised by UV transillumination. Fragments to be sequenced were excised from the gel, DNA was purified using a Bioline gel purification kit (Bioline, USA), eluted in 2×10 µl of elution buffer, and the concentration checked by use of a Nanodrop (Thermoscientific, Wilmington, USA). Approximately 40 ng of cleaned PCR product was then used for direct sequencing with the same primers used for the initial amplification of the product. Products were sequenced using the ABI Big-Dye reaction mix (Applied Biosystems, California) at the Ramaciotti Centre for Gene Function Analysis, University of New South Wales. Resulting sequences were checked using tBlastx analyses against the GenBank database. GenBank accession numbers were: JQ617416–JQ617426.

### Light Microscopy

Motile and non-motile cells were visualised using brightfield and differential interference contrast light microscopy (LM) using a Zeiss Axioskop compound microscope (Zeiss, Munchen-Hallbergmoos, Germany). Micrographs were obtained with an Axiocam digital camera (Zeiss, Munchen-Hallbergmoos, Germany).

### Scanning Electron Microscopy

Dense live culture was dropped onto glass coverslips that were pre-treated with poly-L-lysine. An approximately equal amount of 2% osmium tetroxide was added to fix cells, and left for 20 min. Coverslips were then submerged in distilled water 10 min. Cells were dehydrated by immersion in 10% ethanol for 10 min, followed by 10 min in each of 30, 50, 70, 90 and 100% ethanol. Finally, specimens were critical point dried using liquid carbon dioxide. Coverslips were attached to metal stubs, and sputter-coated with gold-palladium. Images were taken on Zeiss Ultra Plus Field Emission Scanning Electron Microscope (FESEM) at 5–10 kV.

### Transmission Electron Microscopy

The cultured cells were transferred into Eppendorf tubes and concentrated by slow centrifugation (1,500 rpm for 1.5 min). The first fixation step was done by adding 2.5% (v/v) glutaraldehyde (in F/2 medium) on ice for 80 minutes. Two washing steps with F/2 medium followed before post-fixation with 1% (w/v) OsO_4_ (in F/2 medium) for 90 min at room temperature. The sample was then washed twice with distilled water, gradually dehydrated with increasing amounts of ethanol and then infiltrated with propylene oxide-resin mixtures. Finally, the cells were embedded in EMBed 812 resin that was polymerized at 60°C. A diamond knife on a Reichert Ultracut E ultramicrotome was used to cut ultrathin sections, which were then stained with uranyl acetate and lead citrate. The sections were viewed with a Zeiss EM 902A Transmission electron microscope (TEM).

For whole mount preparations, a Pioloform-coated mesh grid was placed on a drop of the culture (cell suspension) for 3 min, removed and placed on a drop of 1% uranyl acetate for about 1 min, removed and rinsed in 4 drops of distilled water. After drying observations were made with a Zeiss EM 902A transmission electron microscope.

### Phylogenetic Analysis

Sequences obtained using the degenerate KS primers were translated and searched against the translated NCBI non-redundant nucleotide database and EST databases, for matches with dinoflagellates or other alveolates. Other sequences for cytochrome *b*, ITS1-5.8s-ITS2 rDNA, and LSU rDNA were obtained using searches on GenBank for sequences of *Amphidinium* and, in the case of cytochrome b, other dinoflagellates. Alignments were performed using ClustalW [31], and checked by hand using Bioedit [32]. FindModel [33] was used to analyse alignments and determine which phylogenetic model to use prior to tree generation. Final alignments consisted of 24 sequences of 335 bp for cytochrome *b*, 17 sequences of 415 bp for ITS1-5.8s-ITS2 rDNA, and 38 sequences of 929 bp for LSU rDNA. Alignments are available by contacting the authors. Maximum likelihood trees were constructed with PhyML [34] using the GTR model with a gamma distribution, which was found to be the most appropriate model for each analysis. One thousand bootstrap replicates were conducted. Bayesian analyses were conducted using the program Mr Bayes 3 [35], using the same optimal model as previously determined. Analyses were run until the average standard deviation of split frequencies was less than 0.01, which consisted of 300,000 generations (for the LSU rRNA alignment) and 1,000,000 generations for the ITS rRNA and cytochrome *b* alignments, sampling every 100 generations. In each case, the potential scale reduction factors (PRSF) were 1.000–1.090. The consensus tree topology of the post burn-in trees and branch lengths were determined. The final phylogenies show the tree topology as determined from the ML analyses, with the branch support as determined by each analysis.

## Results

### Morphology

*Amphidinium massartii* Biecheler 1952: P 24, Figs. 4–5.

Other key references: [17].

Strain CS-259.

**Figure 1:**
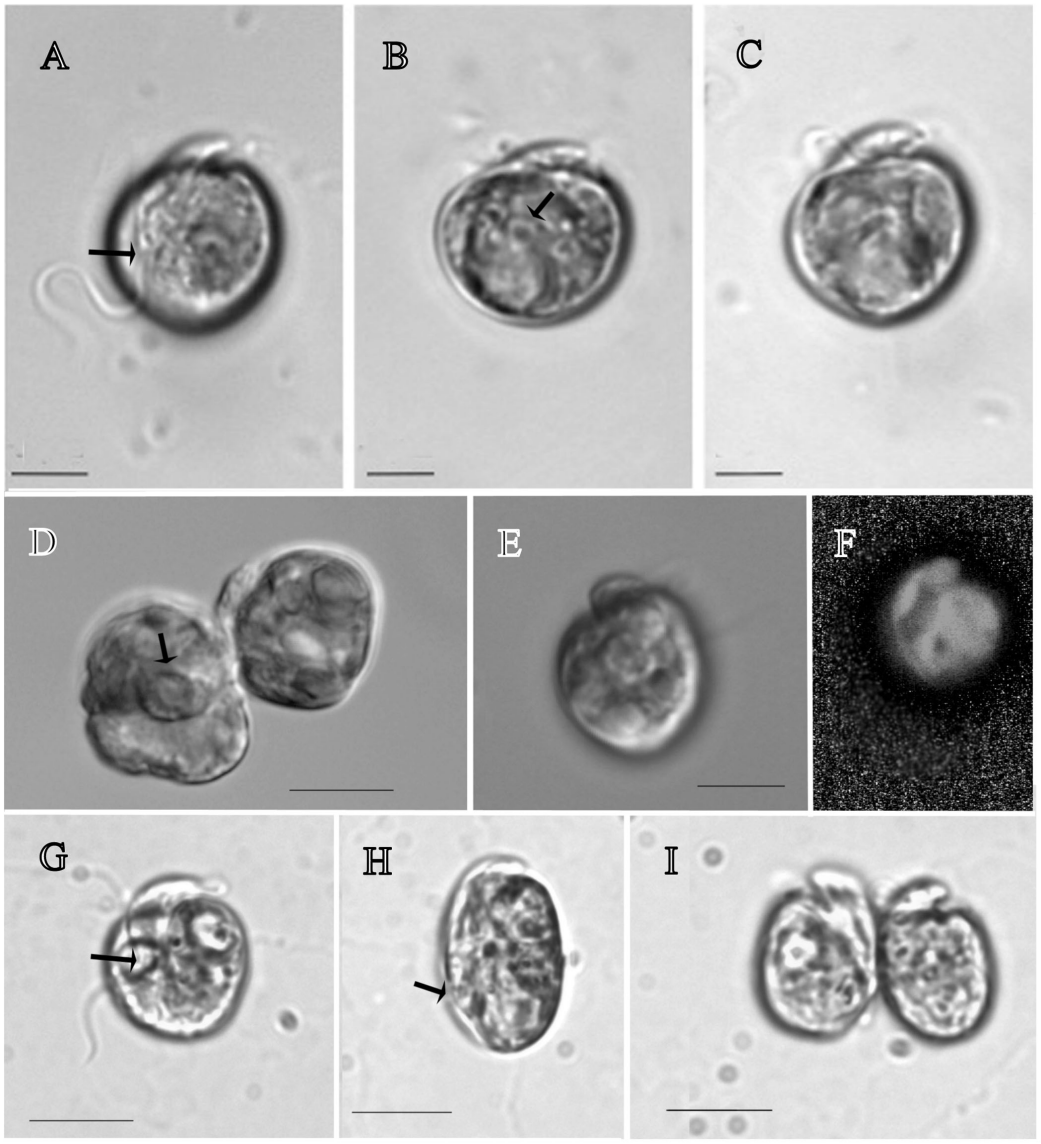
Light micrographs of *Amphidinium massartii* strain CS-259 and *Amphidinium thermaeum* strain CS-109, showing general cell shape, plastid, dividing cells, nucleus, pyrenoid. Scale bars represent 5 µm. (A)–(F), CS-259. (A) *A. massartii* CS-259 in ventral view, showing shape of the epicone and longitudinal flagellum, arrow points to position of flagellar insertion. (B) Low focus image, arrow points to pyrenoid. (C) Cell in dorsal view showing general cell shape, (D) Motile dividing cells, arrow points to starch-sheathed pyrenoid, (E) Cell in lateral view showing flattening, (F) Cell taken using epifluorescent microscopy, showing the plastid with multiple lobes. (G)–(I), CS-109. (G) Cell in ventral view showing general shape and position of flagellar insertion (arrow), (H), Cell in lateral view, arrow points to flagellar insertion, (I), Motile cells shortly following cell division.

**Figure 2:**
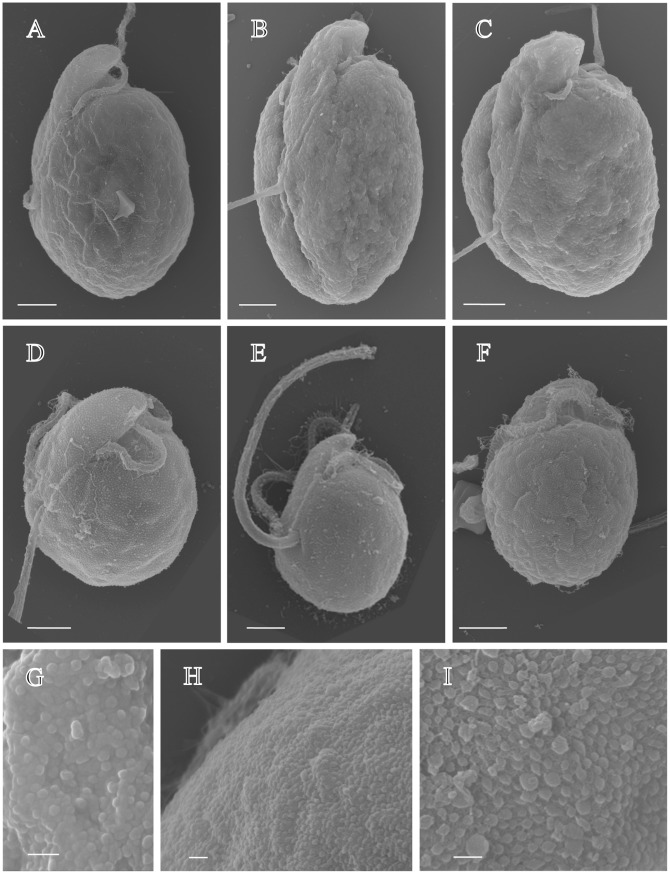
*Amphidinium carterae, A. massartii* and *A. thermaeum* showing position of flagellar insertion, ventral ridge, and gymnodinioid cell surface patterning, taken using the FESEM. (A) *Amphidinium carterae* strain CS-21 (B, C, G) *Amphidinium thermaeum* strain CS-109 (D, E, F, H, I) *Amphidinium massartii* strain CS-259. (A) *A. carterae* strain CS-21, showing the typical morphology of *A.carterae*, including the shorter epicone as compared to *A. massartii*, and the typical gymnodinioid patterning. (B) CS-109, in ventral view, showing general cell shape, the position of flagellar insertion, and ventral ridge. (C) CS-109, showing shape of the epicone and ventral ridge. (D) CS-259 in apico-lateral view, showing ventral ridge and transverse flagellum, (E) CS-259 in lateral view showing wide flagellum, clear lateral ridge, (F) CS-259 in dorso-lateral view, showing gymnodinioid surface patterning. Scale bars represents 2 µm. (G) CS-109, showing high magnification view of cell surface, (H, I) CS-259 showing high magnification view of cell surface. Scale bars represent 200 nm.

**Figure 3:**
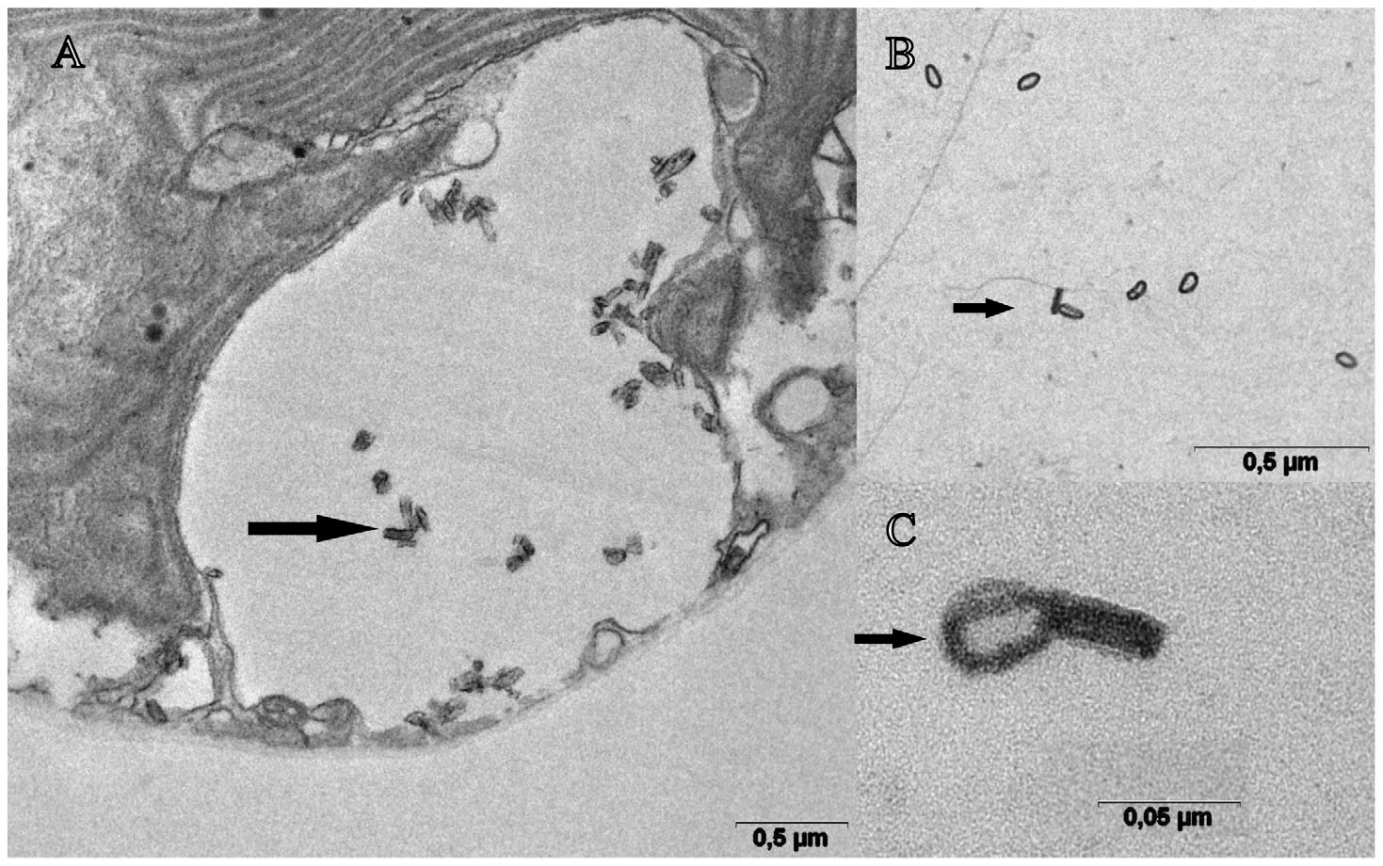
Transmission Electron Microscopy images showing body scales in *Amphidinium massartii*. (A)A section through *Amphidinium massartii* CS-259 showing body scales in alveolae (arrow points to alveolae). (B, C) Whole mount preparation of culture suspension showing the body scales (arrows point to scales).

Cells have a long, narrow epicone and are generally rounded in shape (Fig. 1). Cells are 6.0–12.5 µm in length, (mean 9.5, n = 20), 5.0–11.0 µm in width, (mean 8.2, n = 20), L/W ratio is 0.9–1.6 (Fig. 1A–C). Cells have none to very slight dorso-ventral flattening (breadth - 5 um µm). Cell division by binary fission takes place in the motile cell (Fig. 1D). The longitudinal flagellum is inserted ∼0.6 of the way down the cell. There is a prominent ventral ridge running between the positions of flagellar insertion (Fig. 1A, 2D, E). The longitudinal flagellum is relatively wide, approximately 500 nm (Fig. 2E). The nucleus is rounded, in the posterior of the cell. The gymnodinoid pattern of vesicles can be seen in some cells (Fig. 2F). The plastid is single, with narrow or globular lobes radiating from a central region, and contains a clear ring-shaped starch sheathed pyrenoid of approximately 3 µm diameter (Fig. 1F). Metabolic movement was not observed. Simple body scales were observed as flat, approximately oval-shaped ring-like structures of 45–60 nm in length and 25–30 nm in width (Figs. 3A–C). The natural arrangement of the scales could not be observed. We interpret the irregular accumulations of scales inside alveolar vesicles (Fig. 3A) as a preparation artefact (dislocation).

*Amphidinium thermaeum* Dolapsakis et Economou-Amilli 2009 Figs. 1–10 [36].

Strain CS-109.

Cells are variable in shape, from oval, to rounded or ovoid. Cells are 8.4–16.0 µm in length, (mean 11.3, n = 20), 7.7–12.5 µm in width, (mean 10.0), L/W ratio is 0.9–1.8. Cells are slightly dorso-ventrally flattened (Fig. 1G–H). Motile cells in the process of cell division were observed (Fig. 1I). The longitudinal flagellum is inserted ∼0.6 of the way down the cell (Fig. 1G, H, 2B, C). There is a prominent ventral ridge running between the positions of flagellar insertion (Fig. 2B–C). The nucleus is rounded, in the posterior of the cell. On the cell surface, small rounded structures of approximately less than 100 nm were observed (Figs. 2G–I). The plastid is single, with narrow lobes radiating from a central region, and contains a clear ring-shaped starch-sheathed pyrenoid of approximately 3 µm diameter. Red globules, possibly plastid degradation products, were frequently observed. Metabolic movement was not observed.

The morphology of strains CS-21, CS-383, CS-212 and CS-740 was identical to that described in previous comprehensive descriptions given for *Amphidinium carterae* [17], [37] and is therefore not described in detail here.

### Phylogeny

#### Large subunit rRNA

**Figure 4:**
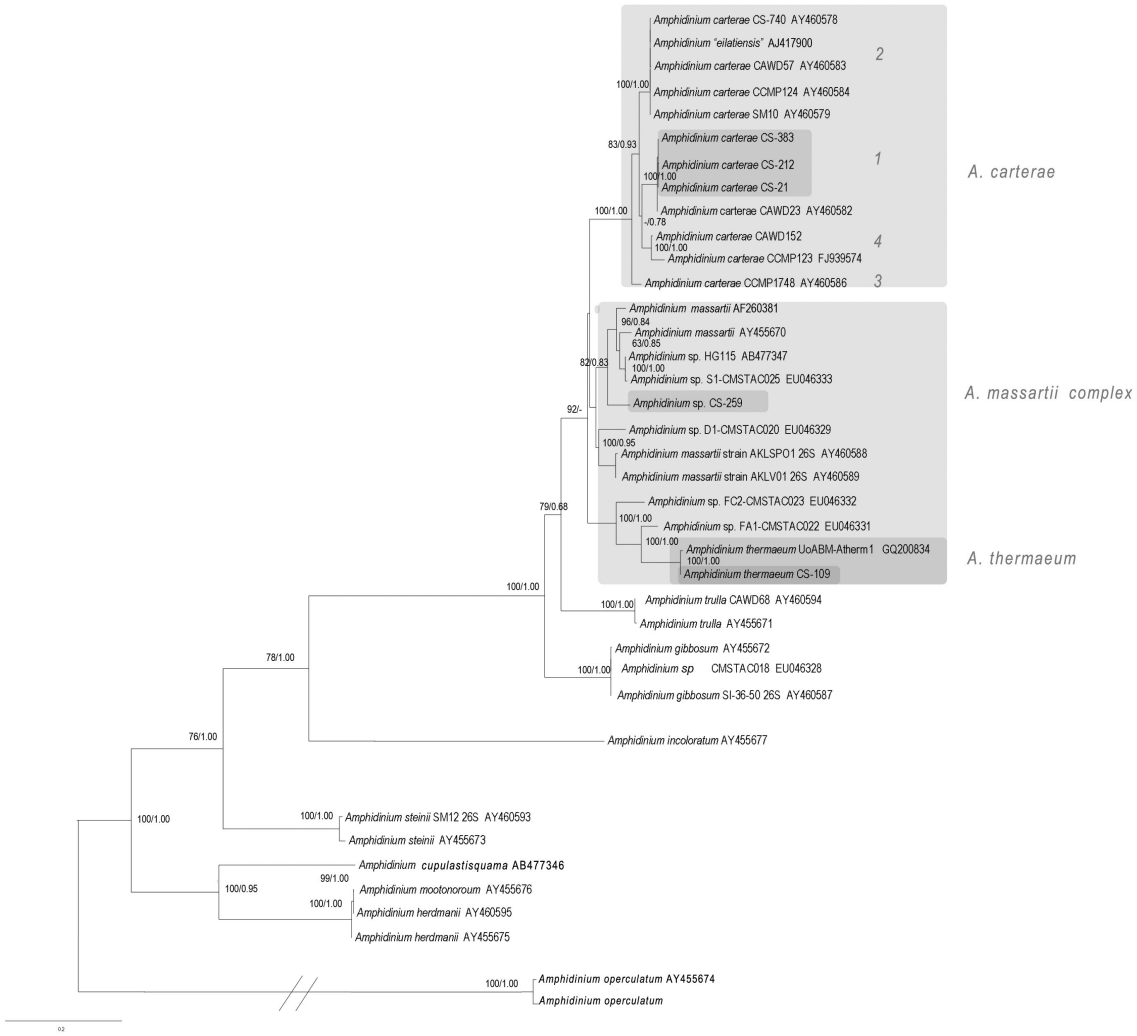
Phylogenetic analysis of alignment of *Amphidinium* partial LSU rDNA sequences (D1–D3 domains), using maximum likelihood. Values at nodes represent bootstrap support values and Bayesian posterior probability support (BS/PP).

The *Amphidinium* strains analysed showed great diversity in LSU rDNA clades (Fig. 4). The *Amphidinium carterae* strains formed a well-supported clade (100/1.00) which differed from the clade containing *Amphidinium massartii* strains by 10.0–11.3% in pairwise comparisons of unambiguously aligned LSU rDNA sequences (930 bp). This was divided into four well supported clades, designated clades 1–4 (Fig. 4). The four clades of *A. carterae* differed from one another by 4.6–6.8%. The *A. carterae* clades 1, 2 and 4 clustered together with reasonable support (83/0.93), while the clade 3 was found to be basal to this clade. Within *A. carterae*, the strains CS-21, 212, and 383, grouped together (Fig. 4, 100/1.00), and differed from each other and other clade 1 *A. carterae* strains by less than 1%, including strains from New Zealand, Denmark, the Caribbean Sea and Belize [17].

*Amphidinium massartii* was found to form 6 clades each with some statistical support (82/0.83–100/1.00, Fig. 4), however, there was no support for the monophyly of this species. The strain CS-259 represented a new unique lineage of the *A. massartii* species complex, differing by 7.4–8.3 % from other strains of *A. massartii*. This strain was the sister group to a clade of three lineages of *A. massartii*, including those previously identified as *A. massartii* clades 1 and 2 (82/0.83, Fig. 4).

*Amphidinium thermaeum* and two other strains (*Amphidinium* sp FC2-CMSTAC023, *Amphidinium* sp. FA1-CMSTAC022) formed a clade with strong support (100/1.00). The strain CS-109 was identified as diverging by less than 1% from the type culture of *A. thermaeum*, isolated from Greece (Fig. 4, 100/1.00, [36]). Two other strains of *Amphidinium* sp. sequences, isolated from the USA, were identified as belonging to this clade (Fig. 4, 100/1.00).

#### ITS1-5.8S-ITS2 rRNA

**Figure 5:**
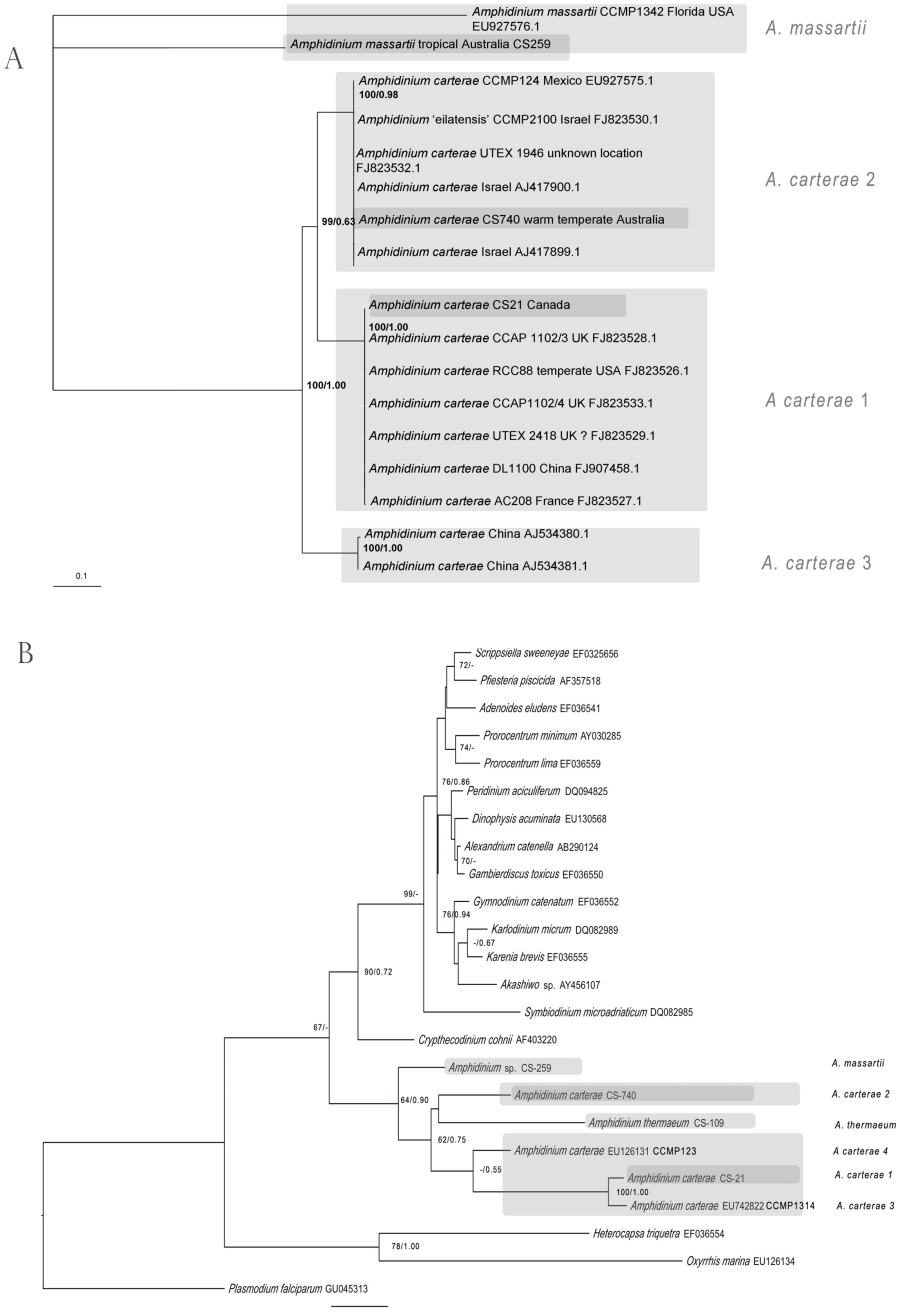
Phylogenetic analysis of alignment of *Amphidinium* species, using maximum likelihood. A) ITS rDNA regions, and B) cytochrome *b* sequences from dinoflagellates, using maximum likelihood. Values at nodes represent bootstrap support values and Bayesian posterior probability support (BS/PP).

The strains of *Amphidinium carterae* and *Amphidinium massartii* analysed showed highly divergent ITS sequences (Fig. 5a), based on pairwise analysis of on average 97 bp of ITS1, 158 bp of the 5.8S region, and on average 160 bp of ITS2. The two strains of *A. massartii*, CS-259 and CCMP1342, differed from each other by 38.8%. The three *A. carterae* clades analysed formed a clear monophyletic group (100/1.00), which differed from the *A. massartii* strains by 33.1–38.1% in aligned sequences. There were three clear clades of *A. carterae ,* which differed from one another by 14.2–23.2% in aligned ITS sequences. Within clade differences in *A. carterae* clades were found to be <0.3% (Fig. 5).

#### Cytochrome *b*

Variation in the 400 bp region of cytochrome *b* amplified in this study, thought to be a relatively conserved region, that was developed for use in dinoflagellate barcoding studies [38], was very high within the genus *Amphidinium* compared to that in other dinoflagellates (Fig. 5b). Strains of *Amphidinium* clustered together in the same clade (64/0.90), however, strains differed from one another by 17–41 %. The species *Amphidinium carterae* was paraphyletic, as a strain of the species *Amphidinium thermaeum* was found to cluster with it, albeit with low support (62/0.75). The three clades of *A. carterae* differed from one another by 20–25%. Even within clade 1, a difference of 5% in primary sequences was found amongst the *A. carterae* strains CS-21 and CCMP1314.

### Polyketide Synthases

**Figure 6:**
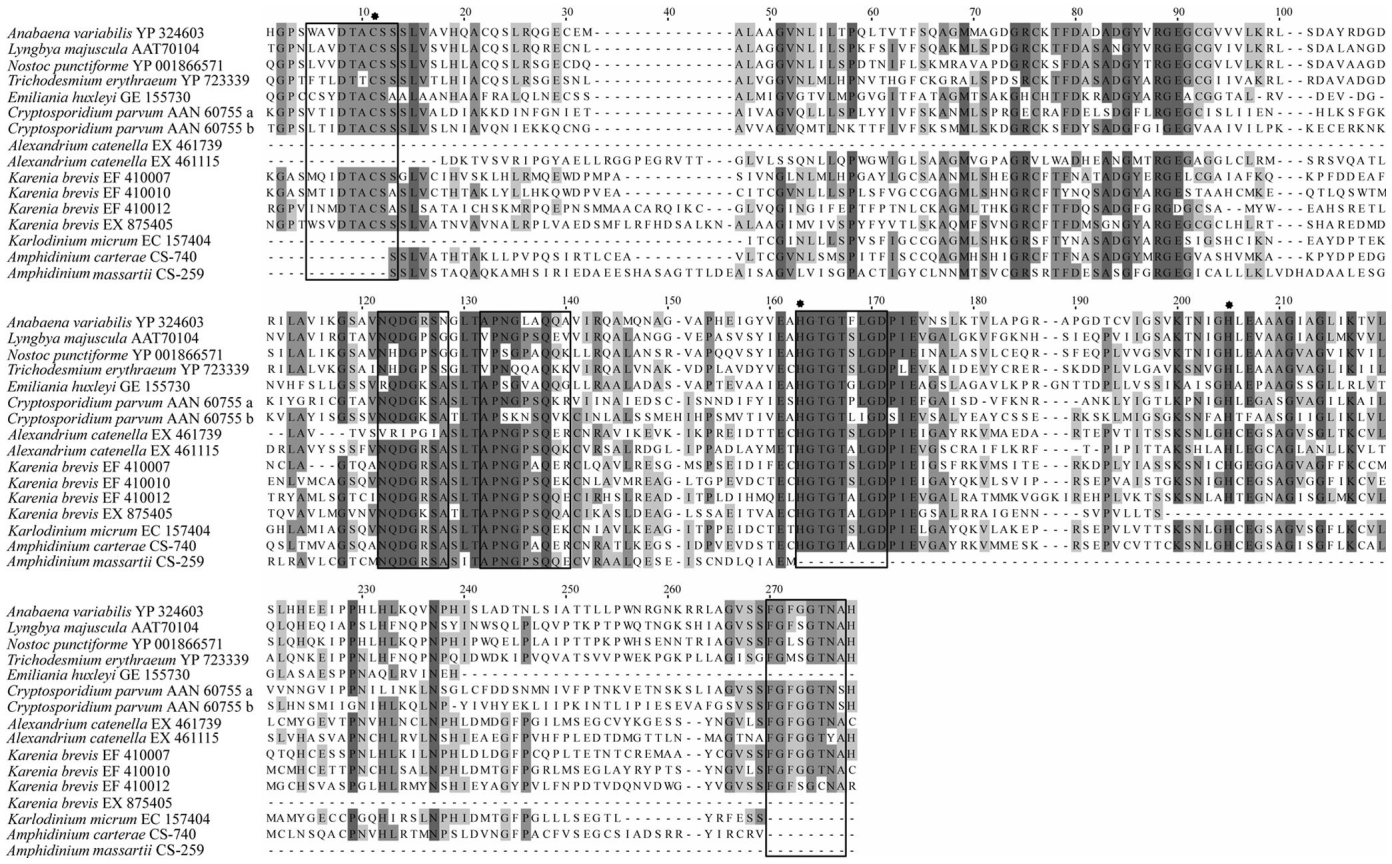
Partial alignment of β-ketosynthase protein sequence from bacteria and alveolates, including three conserved active site residues, and showing conserved regions against which degenerate primers were designed.

Partial KS sequences from *Amphidinium* strain CS-740 (*A. carterae*) and from CS-259 (*A. massartii*) were amplified and sequenced (Table 3, Fig. 6). We attempted to amplify KS sequences from all 6 strains examined in this study (Table 1). The lack of recovery of a KS sequence from a strain is not necessarily indicative of its absence, as even the KS sequences that we did recover varied considerably in DNA sequence, and so the primer sets we used may not have been specific enough to amplify KS genes from every strain. The recovered sequences were included in an alignment of KS sequences from dinoflagellates and several unrelated organisms (Fig. 6). Translated protein sequences were found to align well to sequences from other dinoflagellates, over several key conserved regions (Fig. 6). The *Amphidinium* CS-740 KS sequence was 47% similar to the *Karenia brevis* and *Alexandrium catenella* sequences based on a 233 amino acid alignment; and was found to be most similar to a Type I PKS, which included a dinoflagellate specific spliced leader sequence on the 5′ end (Table 3). The *Amphidinium* CS-259 KS sequence was 32% similar to the *K. brevis* and *A. catenella* sequences based on a 149 amino acid alignment. This sequence was most similar to a PKS sequence from *Karenia brevis*, which had a spliced leader sequence on the 5′ end (Table 3). Two histidine active sites were found to be conserved in the *Amphidinium* CS-740 sequence (Fig. 6).

**Table 3:** Results of tBlastx analysis of putative PKS genes from *Amphidinium* species.

Species and strain number	highest score/E valuein NCBI database	Accession no. oftop contig	Species	Reference
*Amphidinium massartii* CS259	5e^−181^	EF410012.1	*Karenia brevis*	type I polyketide synthase-like protein KB6736 mRNA, (Monroe and Van Dolah 2008)
*Amphidinium carterae*CS740	4e^−74^	EF410010.1	*Karenia brevis*	type I polyketide synthase-like protein KB5361 mRNA (Monroe and Van Dolah 2008)

## Discussion

### Cryptic Species of Dinoflagellates

Until relatively recently, it has been difficult to assess the degree of cryptic diversity present within dinoflagellates. Detailed morphological investigations have not been conducted for most species, so that differences amongst strains thought to represent a single species may have been overlooked. Species misidentifications, leading to incorrectly identified molecular genetic sequences in GenBank and strains in culture collections, have commonly occurred, as in most groups of organisms (i.e. [39]), leading to incorrect conclusions regarding con-specificity or otherwise of strains. Despite this, in the past 10 years, cryptic species have been found within several dinoflagellate taxa that have been well characterised in detailed studies using scanning electron microscopy of multiple strains. Cryptic species were recognised by clear differentiation in ITS or LSU rDNA sequences amongst groups of strains. These include *Scrippsiella trochoidea*, with at least 8 separate clades [6], [40], [41], *Prorocentrum lima* with 3 clades [42], *Alexandrium minutum*, which showed 2 distinct clades [43], *Protoperidinium crassipes*, *P. steidingerae* and *P oblongum*, which each consisted of multiple clades [5], *Peridinium limbaticum* (2 clades, [44]), and *Oxyrrhis marina* (2 clades, [45]).

In this study, very large intraspecific genetic differences were found within the species *Amphidinium carterae* and *Amphidinium massartii*, as well as between species of *Amphidinium* (Figs. 4, 5). The analyses of the mitochondrial cytochrome *b*, ITS rRNA and LSU rRNA sequences each showed the same trend. The intraspecific uncorrected pairwise genetic differences of unambiguously aligned sequences within clades of *A. carterae* and *A. massartii* in LSU rRNA and ITS rRNA were found to be 4.6–8.3%, and 14.2–38.8%, respectively (Fig. 4). Such high diversity in the ITS rRNA gene and LSU rRNA was similarly found in the cytochrome *b* barcoding region (20–25% within the species *Amphidinium carterae*, Fig. 5). As a comparison, in a pairwise comparison of the aligned 440 bp ‘barcoding’ region of cytochrome *b*, species of the family Kareniaceae (*Karenia brevis, Karlodinium micrum*) were found to be only 10–12% different to those of the order Prorocentrales (*Prorocentrum lima, Prorocentrum minimum*, [38], [46]). Therefore, there is a much higher intraspecific diversity within *A. carterae* than between two orders of other dinoflagellate groups [38], [46]. To compare the genetic diversity found in nuclear genes with those found in previous studies of dinoflagellates, Litaker *et al* [47] used uncorrected pairwise differences in ITS rRNA genes to determine the mean divergence between species of dinoflagellates within a genus, and found that differences greater than 4% ( = 0.04 substitutions per site of uncorrected p distances) correlated with a conservative species level difference. As we found intraspecific divergence levels in ITS rDNA within clades of *A. carterae* and *A. massartii* of 4–10 times this level, this would suggest that the clades of *A. carterae* and *A. massartii* represent cryptic species of *Amphidinium*.

### Morphological Comparison of *Amphidinium* Strains

Eukaryotic species designations, including those of dinoflagellates, are currently based on the possession of distinguishing morphological characteristics, which are considered to be indicative of other differences, such as reproductive isolation, in the application of the Biological Species Concept (BSC). The application of the BSC to dinoflagellates is complex, for several reasons including that strains may be homothallic or heterothallic (i.e. [6]). This presents a difficulty when distinguishing species of several genera of dinoflagellates, such as *Symbiodinium*, which are morphologically highly uniform [48] despite high levels of genetic diversity (i.e. [49]), which would indicate likely reproductive incompatibility. Species of the genus *Amphidinium,* as redefined [17], [18] are also morphologically uniform, usually differing only in minor characteristics such as shape, size, and the position of longitudinal flagellar insertion [18], [37]. In particular, the three species *A. carterae, A. massartii* and *A. thermaeum* are highly morphologically similar, overlapping completely in size range and in shape. These three species can be distinguished only on the basis of 1) the shape of the plastid, which is reticulate and distributed throughout the whole cell area in *A. carterae*, and generally more sparse, with several lobes, in *A. massartii* and *A. thermaeum*, 2) the slightly lower position of flagellar insertion in *A. massartii* and *A. thermaeum* compared to *A. carterae* (∼0.6 of the way down the cell, compared to ∼0.4, [17]), 3) asexual division taking place in either cysts or motile cells, and 4) the infrequent observation of metabolic (amoeboid) movement in some cells of *A. thermaeum* [36]. In the present study, the culture CS–109, which was genetically highly similar to sequences from the type strain of *A. thermaeum*, was found to divide in the motile cell, and cell division in cysts was not observed, contrary to the original description of this species [36]. Amoeboid movement was not observed in this strain [36]. This suggests that these morphological characters may not be sufficient to distinguish *A. thermaeum* from *A. massartii*.

Despite this similarity at the LM level, some strains of *Amphidinium* have been found to possess highly unusual ultrastructural characteristics amongst dinoflagellates, such as body scales [22], [50]. These have been found in strains of two species of *Amphidinium,* here considered to be clades of *A. massartii* (HG115 and HG114; [50], as well as *A. cupulatisquama* [22]. The body scales in the two *A. massartii* strains were simple oval rings without a base plate, approximately 65 nm long, 45 nm wide and 13 nm high [50], while those of *A. cupulatisquama* were cup shaped in side view and elliptical in face view, 136 nm long, 91 nm wide and 82 nm high [22], [50]. The scales found in the strain CS-259 most closely resembled those of the two *A. massartii* strains, as they were simple, relatively flat, oval shaped and 45–60 nm in length (Fig. 3). It may be that the possession of body scales is a conserved feature in the species *A. massartii*. The possession of body scales has not been studied for most species or strains of *Amphidinium*. Previously, body scales were not found in thorough ultrastructural studies of clades of *A. carterae* [51]–[53]. However, it is likely that not all clades of *Amphidinium carterae* have been investigated. In addition, other closely related species, such as *Amphidinium trulla,* have not been investigated for the possession of body scales, so it is not possible to determine whether scales are a distinguishing characteristic of *A. massartii* amongst this group of morphologically similar species.

### Polyketide Synthesis in *Amphidinium* Species

The majority of *Amphidinium* strains from which polyketide compounds have been found have not been identified to species level (Table 4). Therefore, we cannot yet determine the phylogenetic distribution of polyketide production among species in the genus *Amphidinium*. In addition, our ability to further investigate the production of these potentially useful compounds (Table 4) is hindered.

**Table 4:** Polyketide compounds isolated to date from strains of species of *Amphidinium*.

Compound name	*Amphidinium* strain from which compound was isolated	Host/origin of *Amphidinium*	Type of polyketide	Toxicity studies	References
amphidinolides (A, B1–B7, C1,C2, D–F, G1–G3, H1–H5, J–S,T1–T5, U–Y)	Y-5, Y-26, Y-42, Y-56, Y-72, Y-100,Y-71, Y-25, HYA002,	flatworm *Amphiscolops* spp, Okinawa	macrolides	cytotoxic against human tumour cell lines- especially amphidinolides B, N,and H	reviewedin [56], [57]
	*A. gibbosum* (S1-36-5)	free-swimming, US Virgin Islands			[70]
caribenolide I	*A. gibbosum* (S1-36-5)	free-swimming, US Virgin Islands	macrolide	strong cytotoxic activity against human colon tumor cell line HCT 116	[71]
amphidinolactone (A, B)	Y-25	flatworm *Amphiscolops* spp, Okinawa	macrolides	modest cytotoxicity	[56], [72], [73]
iriomoteolides (1a-1c, 3a, 4a)	HYA024	benthic *Amphidinium,* Japan	macrolides	strong cytotoxic activity against human colon tumor cell line HCT 116	[26,56,74–76]
amphidinins (A,B)	Y-5, Y-56	flatworm *Amphiscolops* spp, Okinawa	short linear polyketides	moderate cytotoxicity against murine lymphoma L1210 and human epidermoid carcinoma KB cells in vitro	[58], [59]
colopsinols (A–E)	Y-5	flatworm *Amphiscolops* spp, Okinawa	long-chain polyketides	A’ has inhibitory activity against DNA polymerase α and β, ‘C’ and ‘E’ cytotoxic against L1210 cells	[77]–[79]
luteophanols (A–D)	Y-52	flatworm *Pseudaphanostoma luteocoloris,* Okinawa	long-chain polyketides	A’ exhibited weak antimicrobial activity	[80]–[83]
amphezonol (A)	Y-72	flatworm *Amphiscolops* spp, Okinawa	long-chain polyketide	modest inhibitory activity against DNA polymerase α	[84]
amphidinols (1–17)	*A. carterae*	Bahamas	long-chain polyketides	antifungal and hemolytic activity	[85]
	*A. carterae* CAWD 57	New Zealand			[62]
	*A. klebsii* NIES 613	surface of seaweed, Japan			[60,61,63–65]
lingshuiols A,B/ symbiopolyol	*Amphidinium* sp KD-056	jellyfish *Mastigias papua*, Japan	long-chain polyketides	inhibitory activity against the expression of VCAM-1 in human umbilical vein endothelial cells	[86]
	*Amphidinium* sp	China		powerful cytotoxic activity	[87], [88]
karatungiols A and B	*Amphidinium* sp	unidentified marine acoel flatworm, Indonesia	long-chain polyketides	‘A’ has antifungal activity against NBRC4407 *Aspergillus niger* and antiprotozoan activity against *Trichomonas foetus*	[89]
carteraol E	*A. carterae* AC021117009	surface of seaweed, Taiwan	long-chain polyketides	potent ichthyotoxicity, and antifungal activity against *Aspergillus niger,* but not cytotoxic to cancer cells	[90]
amphidinoketides	*A. gibbosum* (S1-36-5)	free-swimming, US Virgin Islands	long-chain polyketides	cytotoxic against human colon tumor HCT116 cells	[91]
unknown	*A. carterae* CAWD 152	surface of seaweed *Halimeda* sp., in Cook Islands	unknown	Crude extracts of *A. carterae* were toxic to mice by i.p. injection	[92]

In this study, we found partial KS sequences in the *Amphidinium carterae* strain CS-740 (clade 2) and in *Amphidinium massartii* strain CS-259 (Table 3, Fig. 6) using degenerate primers designed to target PKS genes from alveolates, as opposed to bacterial or fungal derived PKS genes. To confirm that these sequences were eukaryotic in origin, we conducted a tBlastx search of GenBank, and found that their closest matches were Type I polyketide complete transcripts from *Karenia brevis*, which have been found to have dinoflagellate specific spliced leader sequences on the 5′ end [29].

Prior to this study, polyketide synthase genes had been isolated from a single strain of an unidentified species of *Amphidinium* [15]. That study used degenerate PKS I primers to identify a clone from a genomic DNA library of *Amphidinium* strain Y-42. This clone contained an insert of 36.4 kb, with six open reading frames showing similarity to KS, AT, DH, KR, ACP and TE domains [15] of PKS I genes. However the KS-like genes sequenced from Y-42 are too different at the nucleotide level to be aligned with those from *A. carterae* strain CS-740 and *A. massartii* strain CS-259.

Species of *Amphidinium* have only occasionally been involved in Harmful Algal Blooms (HABs) [54], [55], however they produce a profusion of different types of bioactive compounds, many of which show promise for development as therapeutic agents (Table 4). Polyketides produced by *Amphidinium* species are extremely diverse in structure, and fall broadly into 3 categories: macrolides, short linear polyketides, and long-chain polyketides. Macrolides isolated from *Amphidinium* include amphidinolides, caribenolide I, amphidinolactone, and iriomoteolides. Amphidinolides are the most numerous type of bioactive metabolite found in *Amphidinium*, with 34 different compounds (designated A–H, J–S, T1, U–Y, G2, G3, H2–H5, T2–T5) having been isolated [56], [57]. These compounds were isolated from nine different strains of *Amphidinium*, the majority of which were cultured from cells isolated from marine Okinawan flatworms *Amphiscolops* spp. Amphidinolides have been shown to be cytotoxic against human tumour cells, especially amphidinolides H and N.

Amphidinins A and B are linear short polyketides, isolate from *Amphidinium* strain Y-5, and Y-56 respectively, and exhibit moderate cytotoxicity against murine lymphoma L1210 and human epidermoid carcinoma KB cells [58], [59]. Linear long-chain polyketides isolated from *Amphidinium* spp. include a variety of compounds, the largest group of which is the amphidinols. Amphidinols have been isolated from both an Okinawan strain identified as *Amphidinium klebsii,* and a New Zealand strain of *A. cartera*e clade 2- the same clade from which two of the partial KS sequences in this study were isolated. These polyhydroxy-polyenes have strong antifungal and haemolytic activity [60]–[65], and have shown increase membrane permeability by binding to membrane lipids [60]. A number of other long-chain polyhydroxy compounds similar to amphidinols have also been isolated from various strains of *Amphidinium*. These include lingshuiols, karatungiols, carteraol E, luteophanols, colopsinols, and amphezonol A (Table 4).

### Conclusion

A very high level of cryptic diversity was found to be present within species of *Amphidinium*, including the ‘model dinoflagellate’ *Amphidinium carterae,* as well as *Amphidinium massartii*, corresponding to levels similar to those in distinct genera or families of other dinoflagellates. We found partial ketosynthase sequences from several strains of these species, which may correlate to the propensity for the production of polyketide-related compounds. Given this level of diversity, the precise identification of *Amphidinium* species and clades used in future chemical analysis studies must be done in order to identify novel and potentially useful bioactive secondary metabolites. Future studies with genetically characterised strains of species of *Amphidinium*, and deep sequencing projects, will enable us to determine the genetic basis of the production of particular polyketide compounds and allow an insight into how widespread polyketide production is amongst strains of these cosmopolitan species.
